# Distribution characteristics of integrons and correlation analysis of antibiotic resistance in urine isolated *Enterobacter cloacae*

**DOI:** 10.3389/fcimb.2024.1462742

**Published:** 2024-10-01

**Authors:** Xuedan Qiu, Hui Zhang, Min Jiang, Qiaoping Wu, Qingcao Li, Guangliang Wu

**Affiliations:** ^1^ Department of Clinical Laboratory, The Affiliated Li Huili Hospital, Ningbo University, Ningbo, China; ^2^ Department of Clinical Laboratory, Ninghai County Chengguan Hospital, Ningbo, China; ^3^ Department of Clinical Pharmacy, The Affiliated Li Huili Hospital, Ningbo University, Ningbo, China

**Keywords:** integron, enterobacter cloacae, resistance gene, homology, antibiotic resistance

## Abstract

**Objective:**

This study aims to understand the distribution of integrons among *Enterobacter cloacae* isolated from clinical urine specimens in our hospital, as well as the molecular characteristics of the variable region resistance gene cassette of integron-positive strains and its relationship with drug resistance.

**Methods:**

We collected a total of 80 strains of *Enterobacter cloacae* isolated from urine specimens of hospitalized patients in our hospital between August 2019 and July 2023, and conducted drug sensitivity testing on them. Polymerase Chain Reaction (PCR) technology was employed to screen these strains for Class 1, 2, and 3 integrons. Following this, the promoter and variable regions of integron-positive strains were amplified and sequenced. Additionally, Enterobacterial Repetitive Intergenic Consensus PCR (ERIC-PCR) was utilized for homology analysis of integron-positive strains.

**Results:**

Among the 80 clinical strains, Class 1 integrons were detected in 31 (38.8%) strains, and the following resistance gene cassettes were identified: *aadA2*, *aadA1*, *aadB*, *aac(6’)*, and *catB8*. Three types of variable region promoters were observed: PcS (4 strains), PcW (7 strains), and PcH1 (17 strains), with consistently inactive downstream P2 promoters. Additionally, Class 2 integrons were detected in 5 (6.3%) strains, carrying the variable region resistance gene cassette *dfrA1-sat2-aadA1*. The promoters for Class 2 integrons were uniformly of the Pc2D-Pc2A-Pc2B-Pc2C type. No Class 3 integrons were detected. The strains containing integrons showed significantly higher resistance rates to ciprofloxacin, compound sulfamethoxazole, levofloxacin, gentamicin, amikacin, and tobramycin compared to those without integrons (P<0.05). 35 strains of *Enterobacter cloacae* carrying integrons are primarily classified into three genotypes: A, B, and C. These genotypes are mainly distributed in the urology department and Intensive Care Unit (ICU). The distribution of variable region gene boxes and promoter types is relatively concentrated in the same genotype.

**Conclusion:**

Our study confirmed that *Enterobacter cloacae* isolated from urine samples predominantly carries Class 1 integrons with an extended array of antibiotic-resistant genes. For future research, it is recommended to explore additional resistance mechanisms and evaluate the effectiveness of new therapeutic strategies. Clinicians should be vigilant about the possibility of clonal dissemination and implement enhanced infection control measures in hospital settings.

## 1 Introduction

In recent years, *Enterobacter cloacae* has become increasingly significant as a pathogen in hospital-acquired infections (Girlich et al., 2021), particularly in the context of urinary tract infections (UTIs) (Elbehiry et al., 2024). The widespread use of antibiotics globally has led to a notable increase in the incidence and rapid escalation of antibiotic resistance in *Enterobacter cloacae* (Liu et al., 2021; Stokes et al., 2022), especially among patients with prolonged hospital stays or compromised immune systems. Specifically, *Enterobacter cloacae* has developed resistance to a broad spectrum of first- and second-line antibiotics, including third-generation cephalosporins, carbapenems, aminoglycosides, and quinolones (Oshiro et al., 2020). This extensive multidrug resistance complicates treatment, significantly reduces clinical efficacy, prolongs hospital stays, increases healthcare costs, and limits therapeutic options. Additionally, the resistance of *Enterobacter cloacae* is often linked to the presence of resistance genes and associated enzymes, which diminishes the effectiveness of conventional treatment regimens (Wu et al., 2018). The rapid dissemination of multidrug resistance within *Enterobacter cloacae* poses a formidable challenge to infection control in healthcare settings, underscoring the need for comprehensive research to understand its transmission mechanisms and develop novel therapeutic strategies. Current research on the antibiotic resistance of *Enterobacter cloacae* primarily focuses on specific resistance mechanisms, such as the detection and analysis of extended-spectrum beta-lactamase (ESBL) and AmpC enzyme production (Jin et al., 2018; Berberian et al., 2019). However, these studies often concentrate on isolated resistance genes or specific enzymes, with relatively less attention given to the mechanisms underlying the spread of resistance, particularly those related to integrons and horizontal gene transfer (HGT). Integrons are mobile genetic elements that can capture and integrate multiple resistance gene cassettes, facilitating their rapid dissemination within bacterial populations and across different species, thereby accelerating the spread of multidrug resistance (Ghaly et al., 2022; Wang et al., 2023). Given the critical role of integrons in the transmission of resistance genes, this study aims to explore the relationship between antibiotic resistance and integrons in *Enterobacter cloacae* strains causing UTIs. The research involves a comprehensive analysis of non-repetitive *Enterobacter cloacae* isolates obtained from local hospital urine samples between August 2019 and July 2023. Antibiotic susceptibility was assessed using the instrument method, while PCR amplification and agarose gel electrophoresis were employed to characterize the distribution of integron types, the diversity of gene cassettes in the variable regions, and the patterns of dissemination among different isolates. Furthermore, the study investigates the correlation between integron-positive strains and specific antibiotic resistance phenotypes, aiming to elucidate the potential mechanisms by which integrons contribute to the spread of resistance. The findings of this research will provide valuable scientific evidence to inform the development of effective infection control strategies and antimicrobial policies, ultimately aiming to mitigate the clinical impact of *Enterobacter cloacae*-induced UTIs.

## 2 Materials and methods

### 2.1 Bacterial strains and specimen source

This study collected 80 non-duplicate strains of *Enterobacter cloacae* isolated from urine samples of hospitalized patients at Ningbo Lihuili Hospital between August 2019 and July 2023. Upon further review of the electronic medical records, it was found that all strains were hospital-acquired. This study was approved by the Ethics Committee of Ningbo Medical Centre Lihuili Hospital, Ningbo University (KY2023SL347-01). *Escherichia coli* ATCC 25922 served as the quality control strain for strain identification and antimicrobial susceptibility testing, *Escherichia coli* DH5a was used as the negative control strain for class 1, 2, and 3 integrons, *Proteus mirabilis* 47437 as the positive control strain for class 1 and 2 integrons, and *Serratia marcescens* 37586 as the positive control strain for class 3 integrons. All quality control strains were purchased from the National Center for Clinical Laboratories, Ministry of Health.

### 2.2 Identification of bacterial strains and antibiotic resistance testing

Remove the experimental bacterial strain from the ultra-low temperature freezer, and allow it to thaw. Once thawed, use a sterile inoculation loop to pick the bacterial suspension and perform streaking on blood agar plates (Antu, China) in sequentially numbered sections. Incubate the plates at 37°C in a thermostatic incubator with 5% CO_2_ for 18-24 hours. The identification and antimicrobial susceptibility testing of bacterial strains are conducted according to the standard operating procedure of the VITEK 2 fully automated microbiology analyzer (manufactured by bioMérieux, France) and the Clinical and Laboratory Standards Institute (CLSI) 2023 standards. Firstly, a single colony of the experimental bacterial strain is picked and suspended in sterile water containing 4.5% NaCl to achieve a turbidity of 0.5 McFarland. Subsequently, the bacterial identification card GN and the antimicrobial susceptibility card N13, both provided with the VITEK 2 system, are used for species identification and antimicrobial susceptibility testing, respectively. The sensitivity test results of *Enterobacter cloacae* to antibiotics were all evaluated according to the CLSI 2023 standards.

### 2.3 DeoxyriboNucleic Acid template preparation and screening for integrons, variable regions, and promoters

DNA templates were obtained using a constant temperature metal bath. Premix TaqTM DNA Polymerase (TaKaRa, Japan) was used for PCR amplification of Class 1, Class 2, and Class 3 integrons, while LA Taq DNA Polymerase (TaKaRa, Japan) was used for PCR amplification of the variable regions and promoters of integrons. The relevant primers are listed in **Table 1**. Based on the results of the variable region from integron-positive strains, the sequence of the integrons variable region gene cassette was obtained. Forward primers were designed using the integrase upstream gene sequence, while reverse primers were designed using the sequence of the variable region upstream gene cassette. Negative and positive controls were established for each batch of experiments. The above experimental method has been widely applied in integron-related detection and is widely recognized in the industry (Li et al., 2022; Ma et al., 2022; Zhu et al., 2024).

**Table 1:** The primers and their sequences for integron screening.

Primer	Sequence (5′→3′)	Target gene or region	Reference
intF	CCAAGCTCTCGGGTAACATC	intI1	(Lu et al., 2022)
P2R	GCCCAGCTTCTGTATGGAAC		(Lu et al., 2022)
intI2F	GTAGCAAACGAGTGACGAAATG	intI2	(Lu et al., 2022)
intI2R	CACGGATATGCGACAAAAAGGT		(Lu et al., 2022)
intI3F	AGTGGGTGGCGAATGAGTG	IntI3	(Lu et al., 2022)
IntI3R	TGTTCTTGTATCGGCAGGTG		(Lu et al., 2022)
5CS	GGCATCCAAGCAGCAAG	Class 1 integron variable region	(Lu et al., 2022)
3CS	AAGCAGACTTGACCTGA		(Lu et al., 2022)
INF2	TGGGTGAGATAATGTGCATC	Class 2 integron variable region	(Lu et al., 2022)
INB2	TCGAGAGAGGATATGGAAGG		(Lu et al., 2022)
ERIC2	AAGTAAGTGACTGGGGTGAGCG	ERIC-PCR	(Lu et al., 2022)

### 2.4 Sequencing of integrase, variable region, and promoter

For samples showing clear bands on electrophoresis after amplification of integrons, variable region and promoter, indicating the presence of gene cassettes and corresponding sequences, PCR products from these positive electrophoresis bands were sent to Huada Genomics Company for sequencing analysis. The sequencing results were then compared with BLAST for matching, thereby determining the type of integrons, the types and arrangement of gene cassettes in the variable region, and the type of promoter, among which the integrons type was determined by integrase sequencing results.

### 2.5 ERIC-PCR homogeneity testing

The reaction system for ERIC-PCR consists of 16.25 μL of deionized water, 6.75 μL of RTaq DNA polymerase reaction mix (containing dATP, dTTP, dCTP, dGTP, etc.), 1 μL of ERIC2 primer (**Table 1**), and 1 μL of the prepared template, making a total volume of 25 μL. The reaction conditions were as follows: initial denaturation at 94°C for 4 minutes, followed by denaturation at 94°C for 40 seconds, annealing at 40°C for 1 minute, extension at 72°C for 5 minutes, for a total of 40 cycles, and a final extension at 72°C for 10 minutes (Lu et al., 2022). The positions of positive electrophoresis bands were determined by reference to marker positions, and bands at the same position were considered to represent the same genotype, while bands at different positions were considered to represent different genotypes. The bacterial strains were subjected to similarity analysis using the UPGMA method and Dice similarity coefficient in NTsys2.10e software.

### 2.6 Statistical analysis

WHONET 5.6 software was used for statistical analysis of the antimicrobial susceptibility data of clinical isolates of *Enterobacter cloacae* from our hospital can be conducted. The resistance data were analyzed using the χ2 test in SPSS 25.0, with a significance level of *P*<0.05 indicating statistical significance.

## 3 Results

### 3.1 The results of integrons screening for clinical strains

**Figure 1:**
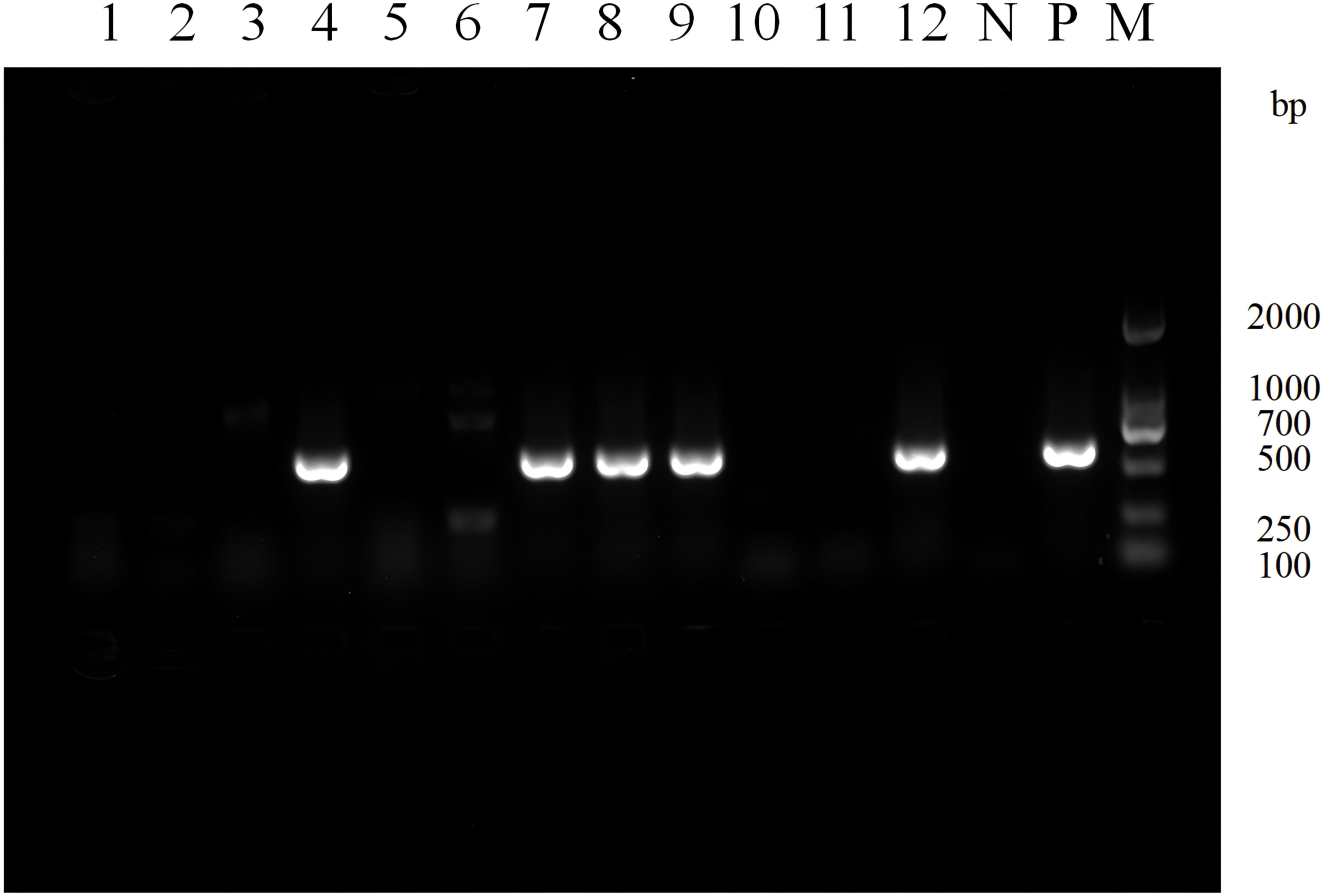
Electrophoresis image of PCR products for screening class 1 integrons in partial experimental bacterial strains. Lane N: Negative control, Lane P: Positive control, Lane M: DNA Marker. Lane 1-12: Strain 30-19. A positive band at 615 bp indicates the presence of class 1 integron.

Among the 80 clinical strains, Class 1 integrons were detected in 31 (38.8%) strains, Class 2 integrons were detected in 5 (6.3%) strains, and no Class 3 integrons were detected. The electrophoresis results of Class 1 integrons amplification products for some clinical isolates are shown in **Figure 1**.

### 3.2 The detection results of the variable region resistance gene cassette

**Figure 2:**
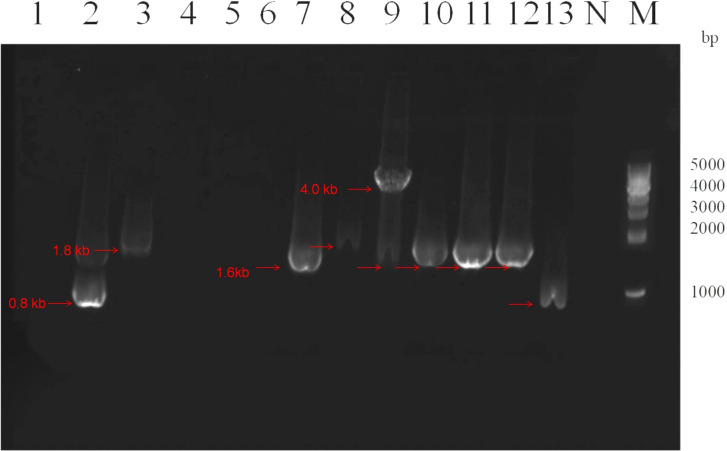
Electrophoresis image of PCR products for variable region of partial class 1 integrons. Lane 1-13: Strains 1, 2, 4, 5, 6, 19, 34, 37, 39, 46, 50, 51 and 70 respectively. Lane N: Negative control, Lane M: DNA Marker. The red arrows indicate amplification products of different lengths for the variable regions of different strains, located approximately at positions 0.8 kb, 1.6 kb, 1.8 kb, and 4.0 kb, respectively. The integron variable regions at the same band position are considered to be of the same size.

The variable region electrophoresis results of 31 Class 1 integron-positive strains showed that 28 strains displayed visible amplification bands in the variable region, while the remaining 3 strains did not show amplification products in the variable region. Among them, there were primarily four different lengths of amplification products, approximately located at positions of 0.8 kb, 1.6 kb, 1.8 kb, and 4.0 kb. For the 5 Class 2 integron-positive strains, the variable region electrophoresis results showed that 4 strains displayed amplification bands in the variable region, while 1 strain did not show amplification products in the variable region. One strain exhibited amplification bands in both Class 1 and Class 2 integrons variable regions. The electrophoresis results of variable region amplification products for some strains are shown in **Figure 2**.

### 3.3 The sequencing results of the variable region amplification products

The amplified products of the variable region for Class 1 and Class 2 integrons were sequenced and compared using BLAST analysis. The results revealed that the Class 1 integrons variable region amplification products could detect 10 types of resistance gene cassettes. The predominant types of resistance genes in these cassettes were associated with aminoglycosides (*aadA2*, *aadA1*, *aadB*, *aac(6’)*) and chloramphenicol (*catB8*). On the other hand, the Class 2 integrons variable region amplification products detected one type of resistance gene cassette, which was *dfrA1-sat2-aadA1*. This cassette confers resistance to trimethoprim, streptothricin, and aminoglycosides (**Table 2**).

**Table 2:** Distribution of class 1 and class 2 integrons and gene cassettes in 35 clinical isolates of *Enterobacter cloacae*.

Number	IntI1	>Variable Region of Class 1 Integron	IntI2	Variable Region of Class 2 Integron
9	+	*catB8-aadA1*	-	ND
5	+	*aadB-aadA2*	-	ND
5	+	*aadA1*	-	ND
2	+	*dfrA16*	-	ND
2	+	*aac(6’)-Ib-cr5-arr-3-dfrA27*	-	ND
2	+	*aac(6’)-Ib-catB8-aadA1*	-	ND
1	+	*aac(6’)-Ib-cr-arr-3*	-	ND
1	+	*aac(6’)-IIc-ereA2*	-	ND
1	+	*catB8-aadA1*	+	*dfrA1-sat2-aadA1-ybeA-ybfA-ybfB-ybgA*
3	-	ND	+	*dfrA1-sat2-aadA1-ybeA-ybfA-ybfB-ybgA*
1	-	ND	+	ND
3	+	ND	-	ND

IntI1, Class 1 Integron; IntI2, Class 2 Integron; ND, no PCR product detected; *dfrA*, trimethoprim resistance gene; *ereA2*, erythromycin esterase gene; sat2, streptothricin resistance gene cassette; aac(6’), aminoglycoside resistance gene; *Ib*, transposon sequence; cr, conferring resistance to fluoroquinolones; *arr-3*, levofloxacin-inactivating gene, *ybeA, ybfA, ybfB, ybgA* are four open reading frames (ORFs), whether they are resistance gene cassettes and their functions remain to be determined.

### 3.4 Promoter types of integrators

**Figure 3:**
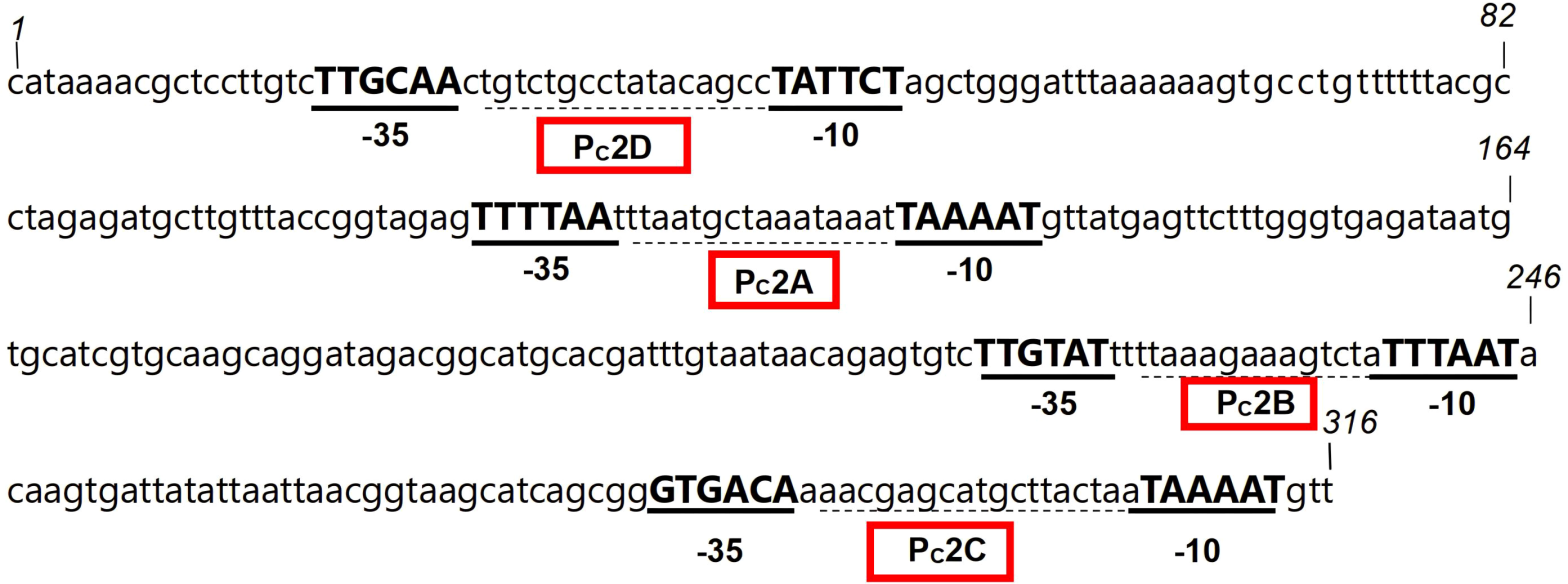
The nucleotide sequence of the promoter in the variable region of the class 2 integron from strain 59. The -35 and -10 elements of the Pc2 promoters are written in bold uppercase, and the Pc2D-Pc2A-Pc2B-Pc2C within the red box is the promoter of the variable region of the class 2 integron.

Sequencing results are analyzed using Vector NTI software to determine the types and quantities of variable region promoters. The Pc promoter detection results in the variable regions of 28 class 1 integron-positive strains are as follows: PcW (weak promoter) in 8 cases, PcS (strong promoter) in 3 cases, and PcH1 (hybrid type 1 promoter) in 17 cases (**Table 3**). The other promoter P2 is an inactive promoter with a 14-base interval between the -35 elements and the -10 elements. The detection results of the variable region promoters in 4 strains positive for class 2 integrons show that they are all Pc2D-Pc2A-Pc2B-Pc2C type, and highly conserved (**Figure 3**).

**Table 3:** Detection results of variable region promoter Pc in 28 class 1 integrase-positive strains.

Promoter types	Arrangement format	Number
PcW (weak promoter)	-10 TGGACA,-35 TAAGCT	8
PcH1 (hybrid type 1 promoter)	-10 TGGACA,-35 TAAACT	17
PcS (strong promoter)	-10 TTGACA,-35 TAAACT	3

### 3.5 The relationship between bacterial strain resistance and integrons

**Figure 4:**
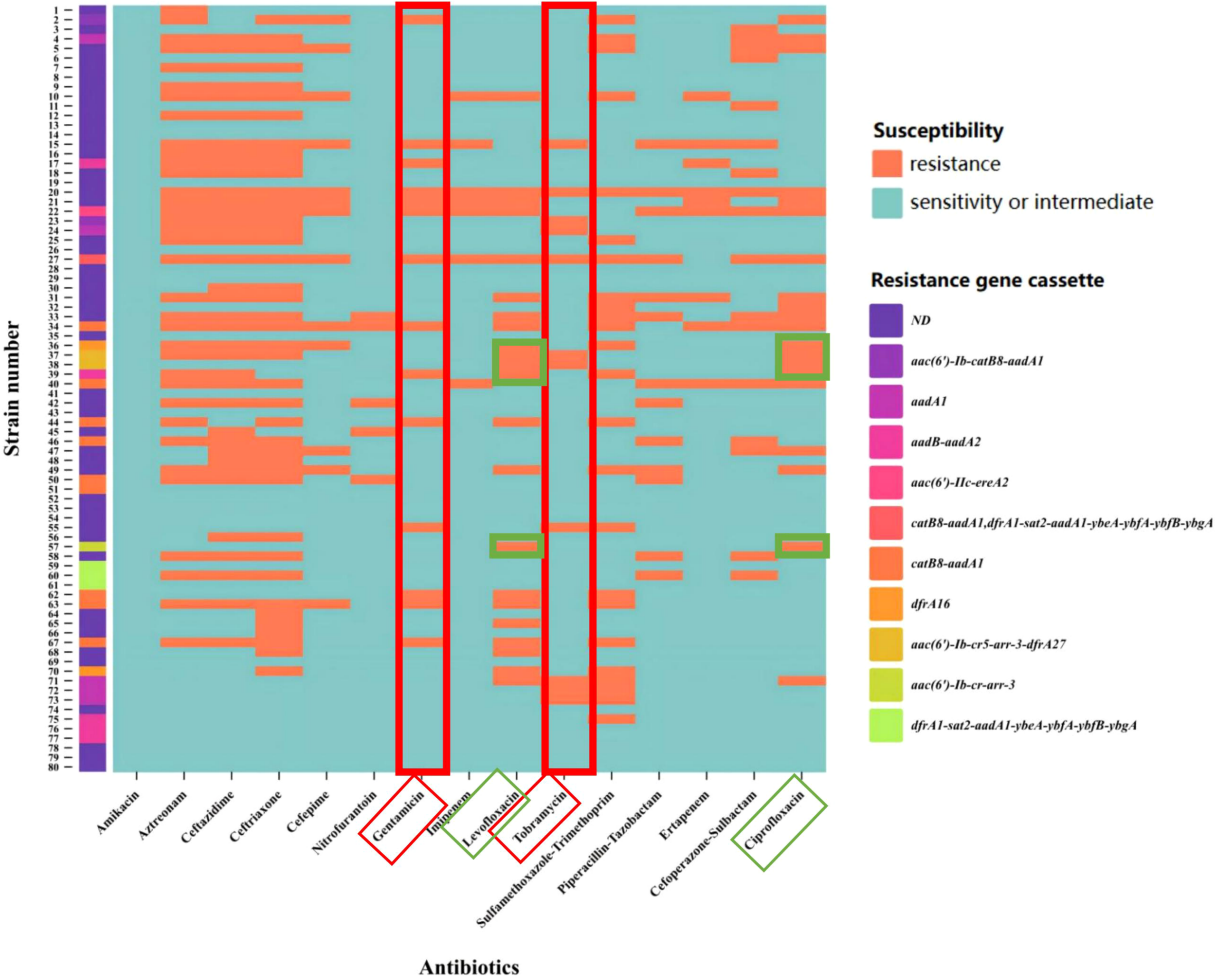
Heatmap of variable region resistance gene cassettes and antibiotic resistance. The X-axis represents antibiotics, and the Y-axis represents strain numbers. the strains carrying the aminoglycoside resistance gene cassette exhibited a higher resistance rate under the pressure of aminoglycoside antibiotics (indicated by the red box). For quinolone antibiotics (indicated by the green box), the experimental results demonstrated that strains 37, 38, and 57 carried quinolone resistance genes and exhibited resistance to both levofloxacin and ciprofloxacin. The colors representing the resistance gene cassettes are shown as illustrated. ND (Not Detected).

*Enterobacter cloacae* exhibited a high level of resistance to aztreonam and cephalosporins. Among the strains resistant to tobramycin and gentamicin, a higher proportion carried aminoglycoside resistance gene cassettes. Additionally, three strains carried fluoroquinolones resistance gene cassettes, and all were resistant to ciprofloxacin and levofloxacin. The specific relationship between variable region resistance gene cassettes and antibiotics is shown in **Figure 4**. Based on statistical analysis, The resistance rates of integron-positive strains to ciprofloxacin, compound sulfamethoxazole, levofloxacin, gentamicin, amikacin, and tobramycin are significantly higher than those of integron-negative strains (*P*< 0.05). The resistance data categorized according to the presence or absence of integrons are summarized in **Table 4**.

**Table 4:** Comparison of antibiotic resistance rates between integrase-positive and integrase-negative strains.

Antibiotics	integrase-positive strains (n=35)		integrase-negative strains (n=45)		*P*
	Number	Resistance rate (%)	Number	Resistance rate (%)	
Ceftriaxone	19	54.3	23	51.1	0.78
Ceftazidime	17	48.6	20	44.4	0.71
Aztreonam	20	57.1	15	33.3	<0.05
Sulfamethoxazole-Trimethoprim	16	45.7	8	17.8	<0.01
Cefoperazone-Sulbactam	8	22.9	9	20.0	0.76
Levofloxacin	14	40.0	8	17.8	<0.05
Ciprofloxacin	13	37.1	7	15.6	<0.05
Cefepime	7	20.0	6	13.3	0.42
Piperacillin-Tazobactam	6	17.1	7	15.6	0.85
Ertapenem	4	11.4	5	11.1	0.96
Gentamicin	10	28.6	4	8.9	<0.05
Imipenem	3	8.6	4	8.9	0.96
Tobramycin	8	22.9	3	6.7	<0.05
Nitrofurantoin	2	5.7	3	6.7	0.86
Amikacin	0	0.0	0	0.0	NA

NA, Not Applicable.

### 3.6 The homogeneity detection results of integron-positive strains

**Figure 5:**
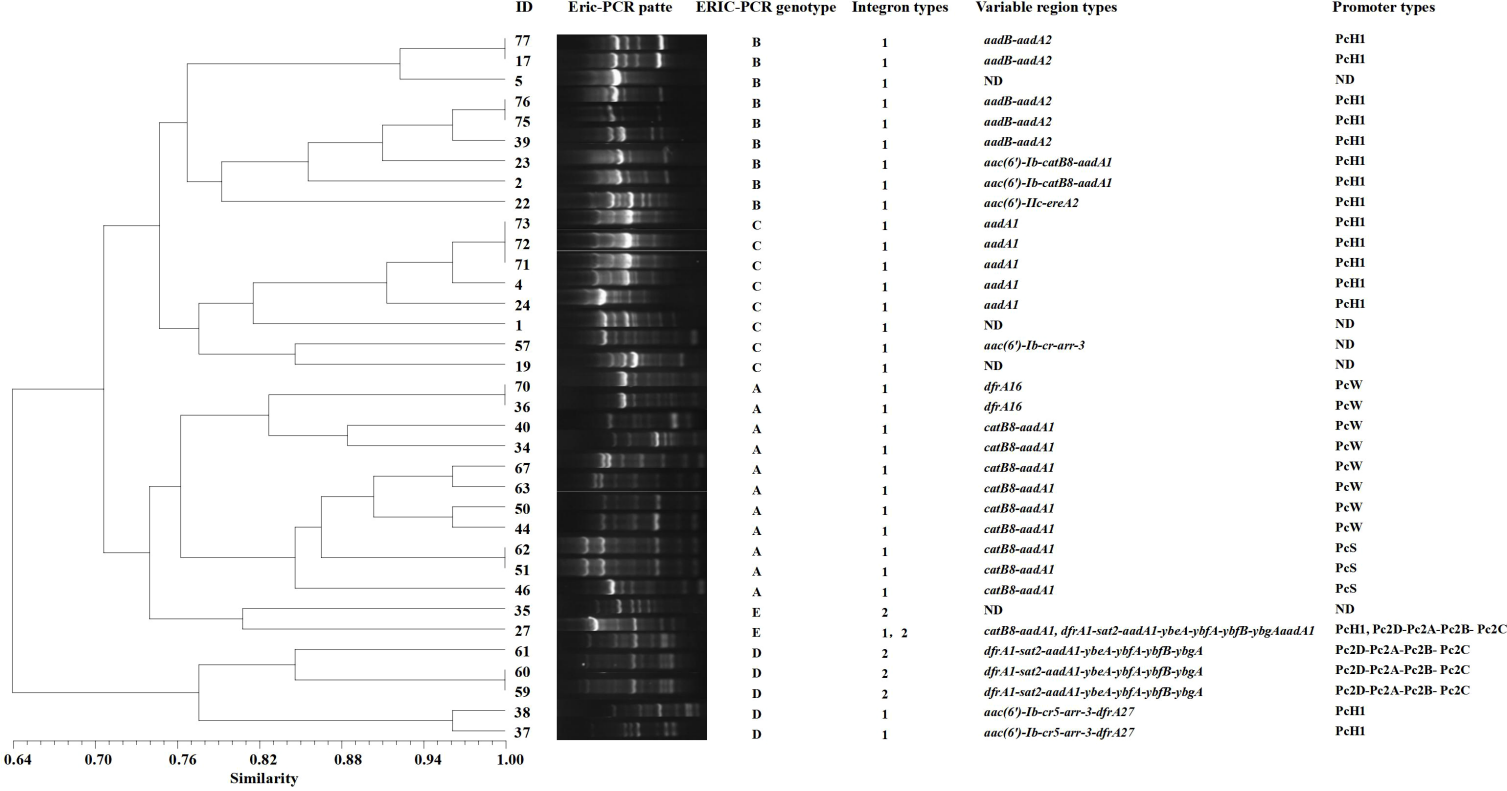
ERIC-PCR genotyping dendrogram of integron-positive strains. Electrophoresis experiments are used to obtain the band positions of different strains. By referring to marker positions, bands at the same location are considered to represent the same genotype, while bands at different positions indicate different genotypes. The Dice similarity coefficient is used to calculate the similarity between strains, which measures the degree of similarity between two strains. The UPGMA method is then applied to transform these similarity data into a phylogenetic tree, ultimately generating a dendrogram that reflects the evolutionary relationships among the strains. ND (Not Detected).

The ERIC-PCR gene typing electrophoresis bands of the 35 integron-positive strains are clear, with the longest product exceeding 3,000 bp and the shortest being less than 650 bp. Using a similarity threshold of greater than 75% to classify strains into the same genotype, the 35 strains can be divided into 5 profiles, designated as type A (31.4%, 11/35), type B (25.7%, 9/35), type C (22.8%, 8/35), type D (14.3%, 5/35), and type E (5.7%, 2/35). The variable region gene cassette *catB8-aadA1* and promoter PcW are mainly concentrated in genotype A, while the variable region gene cassette *aadB-aadA2* and promoter PcH1 are primarily concentrated in genotype B (**Figure 5**). ERIC-PCR genotyping reveals that the same genotypes are more concentrated in the urology department and ICU, whereas they are dispersedly distributed in other clinical departments (**Table 5**).

**Table 5:** Genetic typing and departmental sources of 35 integrase-positive strains.

Department	The number of genotypes					Total number
	A	B	C	D	E	
Urology	5	4	1	1	1	12
Nephrology	1	1	1	1	1	5
ICU	1		2	2		5
Geriatrics	1	1	2			4
Infectious Diseases	1		1			2
Neurology	1		1			2
Adiation therapy		1				1
Orthopedics				1		1
Respiratory medicine		1				1
Hematology	1					1
Neonatology		1				1

## 4 Discussion

In recent years, *Enterobacter cloacae* has become an important pathogen in hospital-acquired infections, particularly in urinary tract infections, with a high incidence rate. According to data published by China Antimicrobial Surveillance Network (CHINET) in 2023, *Enterobacter cloacae* ranks fifth among Gram-negative bacteria in urinary tract infections. Currently, *Enterobacter cloacae* demonstrates an increasing resistance to commonly used antibiotics, including β-lactams, and multidrug resistance is becoming more severe, posing significant challenges to clinical treatment. Multidrug-resistant bacteria outbreaks in hospitals are associated with the transfer of resistance-related genes between strains through transduction, transformation, and conjugation mediated by plasmids, transposons, and other mobile genetic elements. These mechanisms play a crucial role in the emergence and spread of bacterial resistance, with integrons being particularly notable for their ability to transfer resistance genes at the genetic level (Zhang et al., 2020). Integrons are closely associated with bacterial resistance, serving as important mobile genetic elements capable of capturing exogenous genes through site-specific recombination and facilitating their expression, leading to antibiotic resistance in host bacteria. Integrons can reside on plasmids or be part of transposons, participating in the transfer of resistance genes (Macesic et al., 2023), thus facilitating the spread of antibiotic resistance. This study conducted detection of the distribution of integrons and resistance gene cassettes among 80 strains of *Enterobacter cloacae* isolated from clinical urine samples in our hospital over the past five years. Simultaneously, it analyzed the correlation between their presence and the drug resistance of the host bacteria. Furthermore, an analysis and evaluation of the dissemination status of positive strains were conducted.

Integrons are mobile DNA molecules with unique structures capable of capturing and integrating exogenous genes, transforming them into functional gene expression units (Macesic et al., 2023). The detection results revealed that among the 80 strains of *Enterobacter cloacae* screened for integrons, there were a total of 31 strains positive for Class 1 integrons, with a positivity rate of 38.8%. There were 5 strains positive for Class 2 integrons, with a positivity rate of 6.3%. No Class 3 integrons were detected. Our results show a higher prevalence of Class 1 integrons compared to Class 2. This is consistent with studies conducted by (Zhu et al., 2024), which also noted the dominance of Class 1 integrons in similar samples. The detection rates of Class 1 and Class 2 integrons in this study are lower than those reported for other Enterobacteriaceae strains both domestically and internationally (Zhu et al., 2011; Liao et al., 2020; Mirnezami et al., 2020; Fonseca and Vicente, 2022). However, the difference in prevalence may be explained by variations in antibacterial pressure and genetic variability in different regions. In this study, we successfully amplified the variable regions of 28 Class 1 integrons and 4 Class 2 integrons. However, there were still 3 Class 1 integron-positive strains and 1 Class 2 integron-positive strain where the variable regions failed to amplify. This discrepancy may be attributed to genetic recombination or transposon insertional mutations within the integrons variable regions, resulting in the absence of the 3’ conserved segment or excessive length of the variable region gene cassette, surpassing the capability of conventional PCR amplification. This issue warrants further confirmation through subsequent utilization of reverse PCR techniques.

Transcription of genes in bacteria relies on promoters. In Class 1 integrons, most gene cassettes do not contain promoters. Transcription of these gene cassettes relies on the promoter Pc located within the variable region of the 5’ conserved segment of the integrons (Xiao et al., 2019). Studying the types and functions of promoters can further our understanding of the ability and extent to which integrons mediate bacterial resistance. The research findings indicate that variable region promoters are primarily distributed as PcH1 (hybrid 1-type promoter), with PcS (strong promoter) and PcW (weak promoter) being infrequent. Typically, the higher the strength of the promoter, the stronger the expression of the gene cassette (Wei et al., 2013).

The PcH1 detected in this study are hybrid promoters between PcS and PcW, with their transcriptional strength falling between the two. The downstream P2 promoters are all inactive, although they contribute to gene cassette expression at a moderate level. Nonetheless, their capability in integrating and disseminating resistance genes is relatively strong and should be taken seriously. In contrast, the promoters in the variable region of Class 2 integrons are all of Pc2D-Pc2A-Pc2B-Pc2C, highly conserved, consistent with previous reports (Lu et al., 2022).

The primary function of integrons is to store and disseminate gene cassettes. Different integrons carry different gene cassettes, and these cassettes encode various functions (Ma et al., 2022). The resistance genes encoded within these gene cassettes determine the resistance of the host bacteria to specific drugs. The comparative analysis using the Blast database revealed that the variable region of Class 1 integrons in this study harbors a rich diversity of antibiotic resistance genes, totaling over 10 different types. The major antibiotic resistance genes include those conferring resistance to aminoglycosides (*aadA2*, *aadA1*, *aadB*, *aac(6’)*) and chloramphenicol (*catB8*). Additionally, genes conferring resistance to other antibiotic classes such as trimethoprim, erythromycin, fluoroquinolones, and rifampicin were also identified. In contrast, the resistance gene cassettes in the variable region of Class 2 integrons are relatively limited in diversity. The predominant cassette identified is *dfrA1-sat2-aadA1*, which confers resistance to trimethoprim, streptothricin, and aminoglycosides. Through comparative analysis of resistance to common antimicrobial drugs, it was found that integron-positive strains exhibited significantly higher resistance rates to ciprofloxacin, levofloxacin, sulfamethoxazole-trimethoprim, gentamicin, aztreonam, and tobramycin compared to integron-negative strains. Sequence analysis indicates that the variable region of the integrons primarily contains genes conferring resistance to aminoglycosides and sulfonamides. This finding at the genetic level explains the association between integrons and resistance to aminoglycosides and sulfonamides. It aligns with the results of many studies both domestically and internationally (Borruso et al., 2016; Kwiecień et al., 2020). However, the presence of resistance genes carried by integrons cannot fully explain all the observed resistance patterns in the bacterial strains tested in this experiment. This suggests that there are other mechanisms at play, collectively mediating bacterial resistance phenotypes. It is speculated that other resistance genes and mechanisms may exist within the transposons or plasmids where the integrons are located (Xiao et al., 2019).

*Enterobacter cloacae* can easily cause nosocomial infections, is particularly susceptible to clonal spread in urological and ICU wards, and may cause large-scale outbreaks (Klein et al., 2021). It is crucial to use bacterial homogeneity analysis to promptly identify its epidemiological characteristics. Among the various commonly used epidemiological analysis methods, ERIC-PCR technology is characterized by its low cost, simplicity of operation, and good reproducibility (Falah et al., 2019). This study employed ERIC-PCR to perform homogeneity analysis on 35 strains of integron-positive *Enterobacter cloacae*. The results revealed that these 35 strains could be classified into 5 genotypes, primarily A, B, and C. The distribution of the same variable region gene cassette and promoter types within the same genotype indicates a relatively strong homogeneity among *Enterobacter cloacae* strains isolated from our institution. Their distribution was mainly observed in the urology department and ICU, indicating the possible occurrence of clonal dissemination within and between these departments. This may be associated with the characteristics of patients and working environments in the urology department and ICU. Typically, patients in these departments are critically ill and undergo invasive procedures such as urinary catheterization, posing a higher risk of cross-infection (El-Mahdy et al., 2021). Therefore, it is imperative to strengthen disinfection and sterilization of departmental environments and surgical instruments, enhance standardization of medical procedures such as intravenous infusion and urinary catheterization for healthcare workers, to reduce or even prevent cross-infections, and minimize the clonal dissemination of integrons within the hospital.

## 5 Conclusion

In conclusion, our study confirmed that *Enterobacter cloacae* isolated from urine samples predominantly carries Class 1 integrons with an extended array of antibiotic-resistant genes. For future research, it is recommended to explore additional resistance mechanisms and evaluate the effectiveness of new therapeutic strategies. Clinicians should be vigilant about the possibility of clonal dissemination and implement enhanced infection control measures in hospital settings.

## Data Availability

The original contributions presented in the study are included in the article/**Supplementary Material**. Further inquiries can be directed to the corresponding authors.
